# Phosphoproteome profiling reveals critical role of JAK-STAT signaling in maintaining chemoresistance in breast cancer

**DOI:** 10.18632/oncotarget.21801

**Published:** 2017-10-10

**Authors:** Augusto S. Nascimento, Luisa L. Peres, Alessandra V.S. Fari, Renato Milani, Rodrigo A. Silva, Celio Jr. da Costa Fernandes, Maikel P. Peppelenbosch, Carmen V. Ferreira-Halder, Willian F. Zambuzzi

**Affiliations:** ^1^ Bioassays and Cell Dynamics Laboratory, Department of Chemistry and Biochemistry, Bioscience Institute, UNESP, Botucatu, Sao Paulo, Brazil; ^2^ OncoBiomarkers Research Laboratory, Department of Biochemistry and Tissue Biology, Biology Institute, UNICAMP, Campinas, Sao Paulo, Brazil; ^3^ Department of Gastroenterology and Hepatology, Erasmus MC, University Medical Center Rotterdam’s Gravendijkwal 230, NL-3015 CE Rotterdam, The Netherlands

**Keywords:** breast cancer, chemoresistance, phosphoproteome, kinase, JAK

## Abstract

Breast cancer is responsible for 25% of cancer cases and 15% of cancer death among women. Treatment is usually prolonged and hampered by the development of chemoresistance. The molecular mechanisms maintaining the chemoresistant phenotype remains, however, largely obscure. As kinase signaling in general is highly drugable, identification of kinases essential for maintaining chemoresistance could prove therapeutically useful. Hence we compared cellular kinase activity in chemotherapy resistant MCF7Res cells to chemotherapy-sensitive MCF cells using a peptide array approach that provides an atlas of cellular kinase activities and consequently, predominant pathways can be identified. We observed that peptides phosphorylated by elements of JAK-STAT signaling pathway and PKC signaling pathways are subject to extensive kinase activity in MCF7Res cells as compared to chemotherapy-sensitive MCF cells; and Western blotting confirmed relatively strong activation of these signaling pathways in chemoresistant cells. Importantly, treatment of cells with Tofacitinib, a FDA-approved JAK inhibitor, converted chemoresistant cells to chemosensitive cells, inducing apoptosis when used in conjunction with doxorubicin. Thus our results reveal that chemoresistance in breast cancer is associated with activation of JAK/STAT signaling and suggest that JAK2 may be useful for combating chemoresistance in breast cancer.

## INTRODUCTION

It has been estimated that annually 1.7 million new cases of breast cancer are diagnosed around the world [1]. In general, breast cancer is one of the most prominent causes of cancer-related deaths among women, amounting to 25% of cancer cases and 15% of cancer deaths in females [2]. Despite advances in therapy, clinical outcome of breast cancer thus remains unsatisfactory. It is hoped that the unrelenting advance in knowledge of the molecular basis of breast cancer will ultimately reveal novel molecular targets for therapy [3], but currently the main challenge in combating breast cancer lies in the development of chemotherapeutic resistance following the xenobiotic administration that characterizes contemporary treatment protocols. In molecular terms this chemoresistant aspect of breast cancer is denominated as the MDR cell phenotype [4]. As MDR is multifactorial in nature, the MDR phenotype has been associated with multiple cellular changes, including changes in cell cycle patterns, increased efficiency of DNA repair, reduction of apoptotic propensity and modification of drug metabolism [5]. Although the mechanisms mediating the MDR phenotype remain only partly resolved, the notion that activation of specific signaling pathways, *e.g*. PI3K/AKT or Ras/ERK pathway maintain this phenotype has been gaining ground [6, 7, 8]. In breast cancer, however, candidate pathways mediating the MDR phenotype have not been conclusively identified, hampering rational treatment of disease.

Identifying pathways mediating MDR in cancer cells has been traditionally hampered the necessity of pursuing one potential biochemical element involved in this process at a time. Classically, the study of different signaling pathways typically occurs by analyzing the phosphorylated/dephosphorylated amino-acid residues of proteins, which is basically conducted by western blotting approaches. However, this technique is labor intensive and does not allow the analysis of multiple proteins simultaneously and therefore it is limiting for clinical studies and also makes it time consuming to identify potential new molecular targets [9, 10]. Novel therapeutic approaches, however, provide potential for progress in this regard. Especially kinome profiling using peptide arrays allows the simultaneous analysis of over 1000 substrates in a single experiment thus creating more-or-less comprehensive descriptions of cellular global kinase activity [11]. This technology is now revolutionizing signal transduction research and has been successfully employed for kinase activity profiling, biomarkers identification, cell surface marker/glycosylation profile, clinical diagnosis and environmental and food safety analysis [12, 13]. This powerful approach has however not been exploited to obtain insight into the biochemical pathways maintaining the MDR phenotype in cancer. The clinical problem posed by chemoresistance in breast cancer, however, urges of application of this technology for especially this disease.

The above-mentioned considerations prompted us to contrast the kinome of chemotherapy sensitive and chemotherapy-resistant MCF cancer cells. Importantly, we validated the kinome profiling results by western blotting technology applied to identify the involvement of PKCs isoforms. Conversely, we observe that chemoresistance in breast cancer is associated with activation of JAK2-STAT pathways and that inhibition by using a FDA-approved inhibitor (Tofacitinib) of the latter pathway reverts chemoresistant cancer cells to a chemosensitive phenotype, provoking apoptosis when used in combination with doxorubicin. Thus, our results showed that JAK2 inhibition is a rational strategy to combat chemoresistance in breast cancer.

## RESULTS

**Figure 1:**
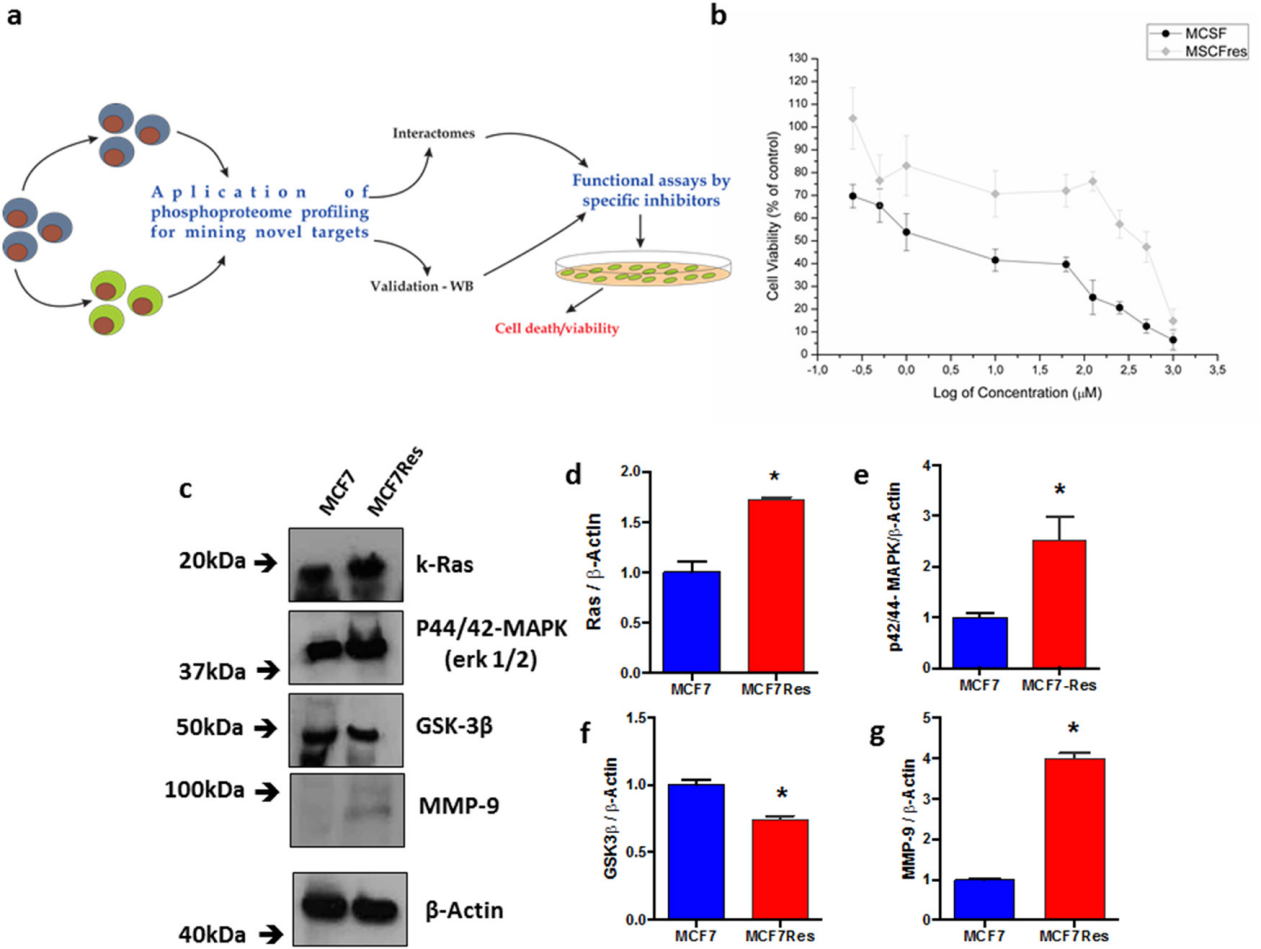
Cellular viability assessed by the mitochondrial function, differential expression of Ras, p44/42-mapk, GSK3ß and MMP-9 **(a)** Schematic depiction of the experimental design employed for this study; **(b)** a dose-response curve to drug treatment - Cytotoxicity of doxorubicin at various concentrations for MCF7 or MCF7Res cells was determined following 24h drug treatment as described in Methods. The means of at least three independent experiments are plotted. Cell viability was assessed by the ability of cells to reduce MTT salt (vital stain) as described in Materials & Methods. Our results show a difference in the viability of the tested lineages. Cells were cultured under routine classic conditions. In the semi-confluence, the cells were lysed using standard lysing buffer (described in M&M) and resolved on SDS-PAGE gel following transfer to PVDF membranes and staining using specific primary antibodies; **(c)** representative blots following probing for Ras, p44/42-mapk (Erk 1/2), GSK3ß and MMP-9 are shown, as well as their arbitrary values obtained by densitometric analysis; (**d-g**, respectively) normalized to the average values of the respective bands of ß-Actin for the appropriate conditions. # means statistical significance (test t-student). The ß-Actin was used as loading sample (approximately 75μg of protein per lane). The differences were significant when ^*^p<0.05.

Firstly, we confirmed that our experimental set up allows investigation of chemoresistance in breast cancer. To this end we compared doxorubicin dose-responses curves of MCF7 cells to MCF7Res cells with respect to the ability of viable cells to reduce MTT salt (vital stain, experimental flow in Figure 1a). The results show that whereas the maternal MCF7 cell line is highly sensitive to doxorubicin (displaying an apparent of 10 μM – 62.5 μM), the MCF7Res culture exhibits an IC_50_ between 500 μM to 1000 μM (Figure 1b). We thus concluded that our setup was suitable for investigating the molecular basis of chemoresistance.

Encouragingly, a preliminary comparison of the maternal MCF7 cell line with MCF7Res cells for signaling elements generally associated with chemoresistance showed that the chemoresistant phenotype was associated with increased expression of p21Ras and MMP9 and reduced expression of GSK3ß, whereas increased phosphorylation was noted of p44/42-MAPK (Figure 1c-1g). Thus biochemical differences between the resistant phenotype and chemosensitive manifestation of MCF7 cells exist and experiments were initiated to identify the underlying signal transduction events.

### A kinome fingerprint of chemoresistant breast cancer

**Figure 2:**
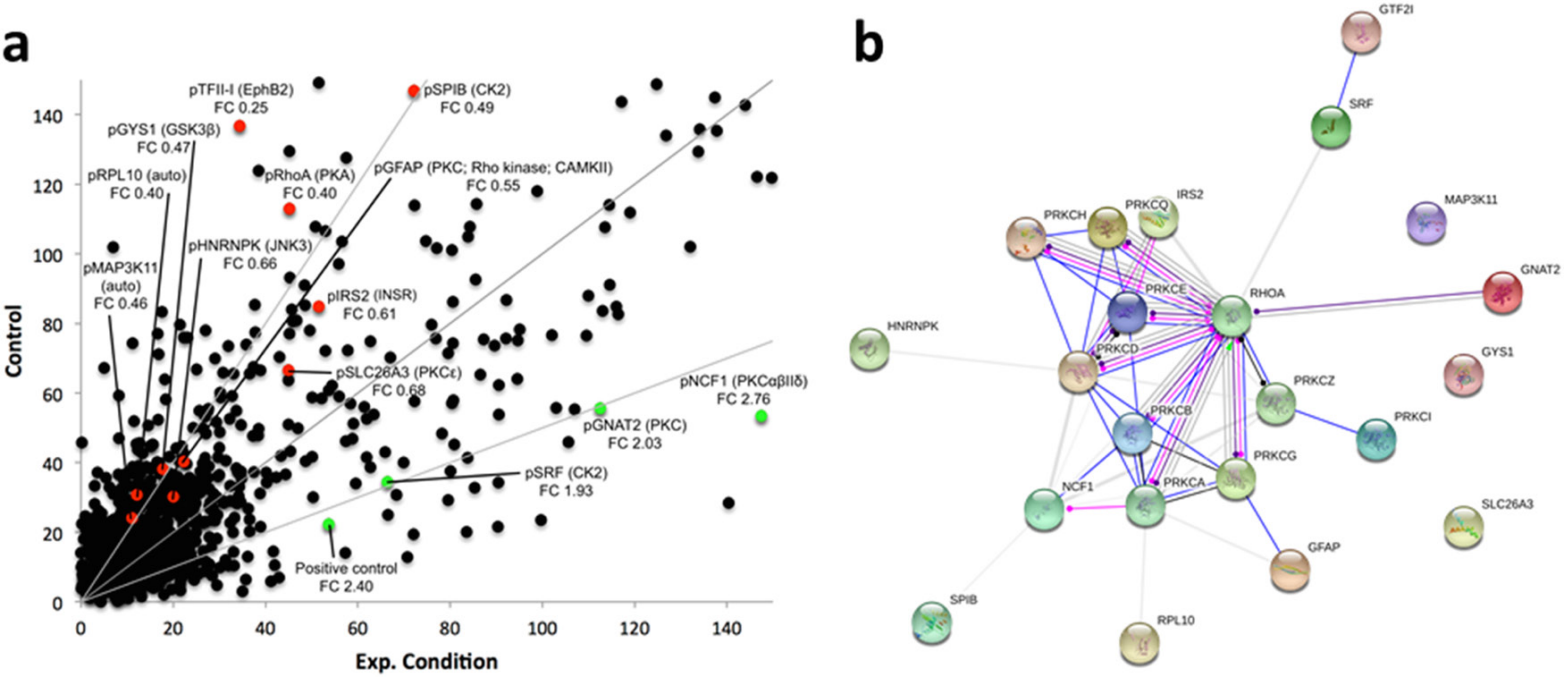
**(a)** Median phosphorylation intensity for all 1024 PepChip kinases and substrates. Colored spots signify individual peptide phosphorylations that exhibit statistically significant differences between the experimental conditions (p<0.05). Red means down regulation of phosphorylation (FC<0.75) whereas green indicates an upregulation of phosphorylation (FC>1.5) in the experimental sample as compared to the control sample. Grey lines represent FoldChange = 0.5; 1; 2, top to bottom. Spots are identified by the phosphorylated substrate (denoted by “p”), followed by the kinase putatively responsible for the phosphorylation event. FC indicates the individual fold change for each colored spot. **(b)** Protein network depicting interactions among protein kinase C variants and proteins found with altered phosphorylation profiles. Blue lines indicate binding, green lines indicate activation and red lines indicate inhibition, whereas purple lines indicate catalysis, pink lines indicate posttranslational modification, yellow lines indicate transcriptional regulation and black lines indicate a generic reaction. Arrows indicate a positive action, bars indicate a negative action and ball-ends indicate a directional interaction of unknown nature. Proteins are represented by gene names. The network was generated by STRING 10.0 [19].

We contrasted the kinome of the maternal chemosensitive MCF7 cell line to that of the chemoresistant MCF7Res cultures. To this end cultures were lysed and resulting lysates were used for *in vitro* phosphorylation of peptide arrays exhibiting more as thousand different kinase substrates. Our results show that the kinome of chemoresistant cells is markedly different from chemosensitive cultures (Figure 2a; Supplementary Table 1). Interesting substrates differentially phosphorylated include GNAT2, NCF1 and SRF that are subject to phosphorylation by PKC isoforms and Casein Kinase 2 (CK2) respectively. A network analysis confirms a central role for PKC variants (Figure 2b). We concluded that chemoresistance of breast cancer cells is associated with a specific kinome profile in which PKC activation has a prominent role.

### PKCs isoform are both differentially expressed and phosphorylated in breast cancer chemoresistant phenotype

**Figure 3:**
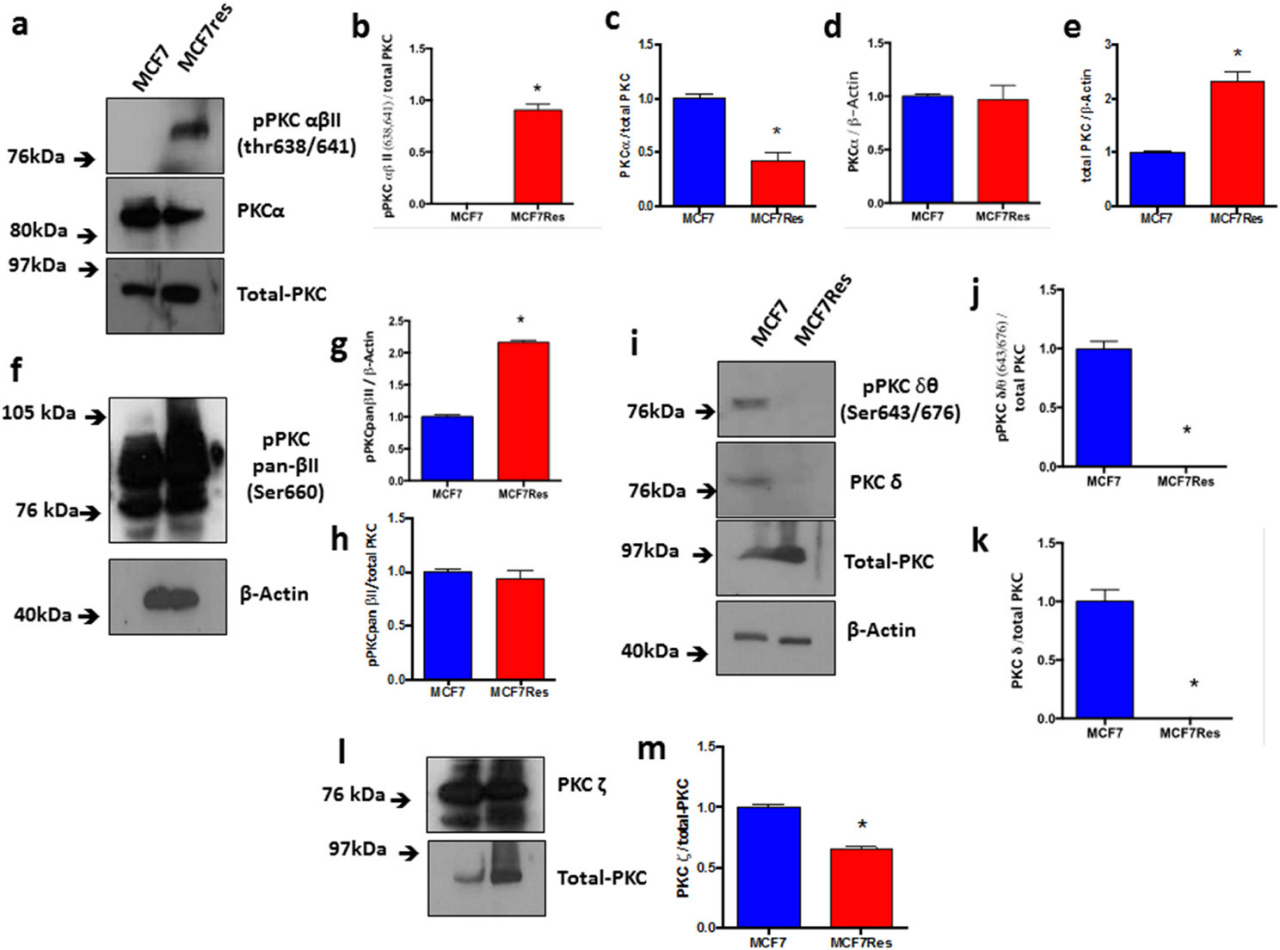
Expression and differential phosphorylations of PKCα, pPKCαβII, total-PKC, pPKC pan-βII, PKCδ, pPKCδθ and PLCγ The cells were cultured under routine classic conditions. In the semi-confluence, the cells were lysed using standard lysing buffer (described in M&M), and proteins were resolved on SDS-PAGE gel and after PVDF membrane transfer, interrogated for expression using specific primary antibodies. ß-Actin was used as loading control (approximately 75 μg of protein per lane). **(a)** Representative blotting for total-PKC, PKCα and pPKCαβII proteins (a), and respective arbitrary values obtained by densitometric analysis normalized by the loading controls **(b-e)**. **(f)** Representative blotting for pPKCαβII and respective arbitrary values obtained by densitometric analysis and normalized by the average values of the respective total-PKC and ß-Actin bands **(g, h)**. **(i)** Representative blottings for pPKCδθ and PKCδ proteins. Graphs **(j)** and **(k)** represent arbitrary values obtained by densitometric analysis of pPKCδθ bands normalized by the average values of the ß-Actin and total-PKC bands, respectively. When normalized by total-PKC, the proteins PKCδ and pPKCδθ were differentially expressed between the two lineages studied. **(l)** Representative blotting of PKCζ protein. Graph **(m)** represents arbitrary values obtained by densitometric analysis of PKCζ bands normalized by the average values of the total-PKC bands. When normalized by total-PKC, the protein PKCζ was differentially expressed between the two lineages studied. Differences were considered significant when ^*^p<0.05.

**Figure 4:**
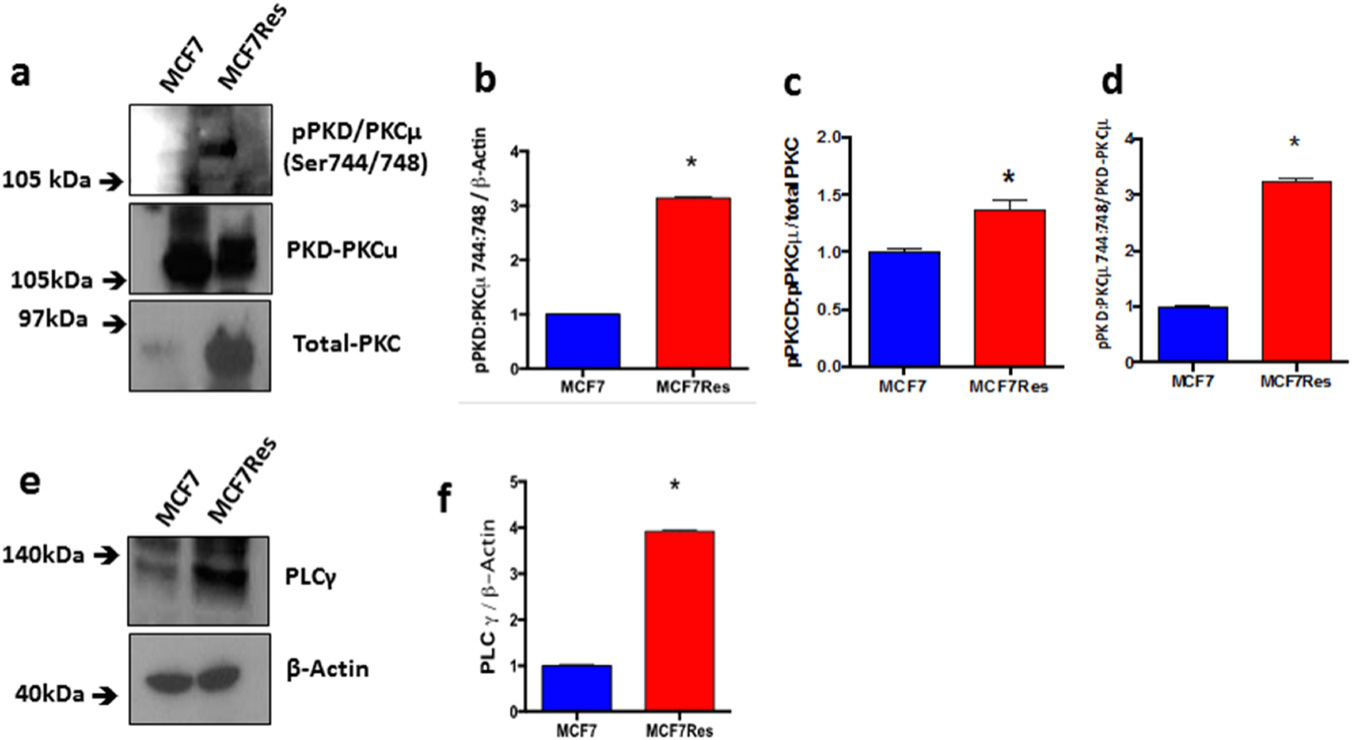
Expression and differential phosphorylation of PKD/PKCμ and PLCγ **(a)** Representative blotting of pPKD/ PKCμ and PKD/PKCμ proteins. Arbitrary values obtained by densitometric analysis of pPKD/PKCμ and PKD/PKCμ bands normalized **(b-d)**. **(e)** Representative blotting of PLCγ protein. Graph **(f)** represents arbitrary values obtained by densitometric analysis of the PLCγ bands normalized by the average values of the ß-Actin bands. When normalized by ß-Actin, the protein PLCγ presented significant difference between the two lineages studied. Differences were considered significant when ^*^p<0.05.

Independent confirmation for the notion that chemoresistance of breast cancer cultures is associated with activation of PKC was obtained from experiments in which we directly assessed both PKC expression and phosphorylation of different PKC isoforms including PKCα, pPKCαβII, pPKC pan-βII, PKD/PKCμ, pPKD/PKCμ, PKCζ, PKCδ, pPKCδθ as well as total PKC (Figure 3). The results show differential PKC activation/inactivation in chemoresistant breast cancer cells, in which especially the phosphorylations of pPKCαβII (Thr638/641) (Figure 3a, 3b) and pPKD/PKCμ (Ser744/748) (Figure 4a-4d) is significantly up-regulated, whereas the phosphorylation of atypical pPKCδθ (Ser643/676) (Figure 3i, 3j) is significantly and substantially reduced. In line with its canonical mode of action, expression of PLCγ (that activates classical PKCs by liberating diacylglycerol from biological membranes) was strongly upregulated in chemoresistant cells (Figure 4e, 4f). The only exception to the activation of classical PKCs came appeared to be PKCβII as an anti- PKC pan-βII (Ser660) antibody failed to detect differences between the groups (Figure 3f-3h) and hence chemoresistance of breast cancer cells involves prominent activation of classical PKCs.

### Detailed network analysis

**Figure 5:**
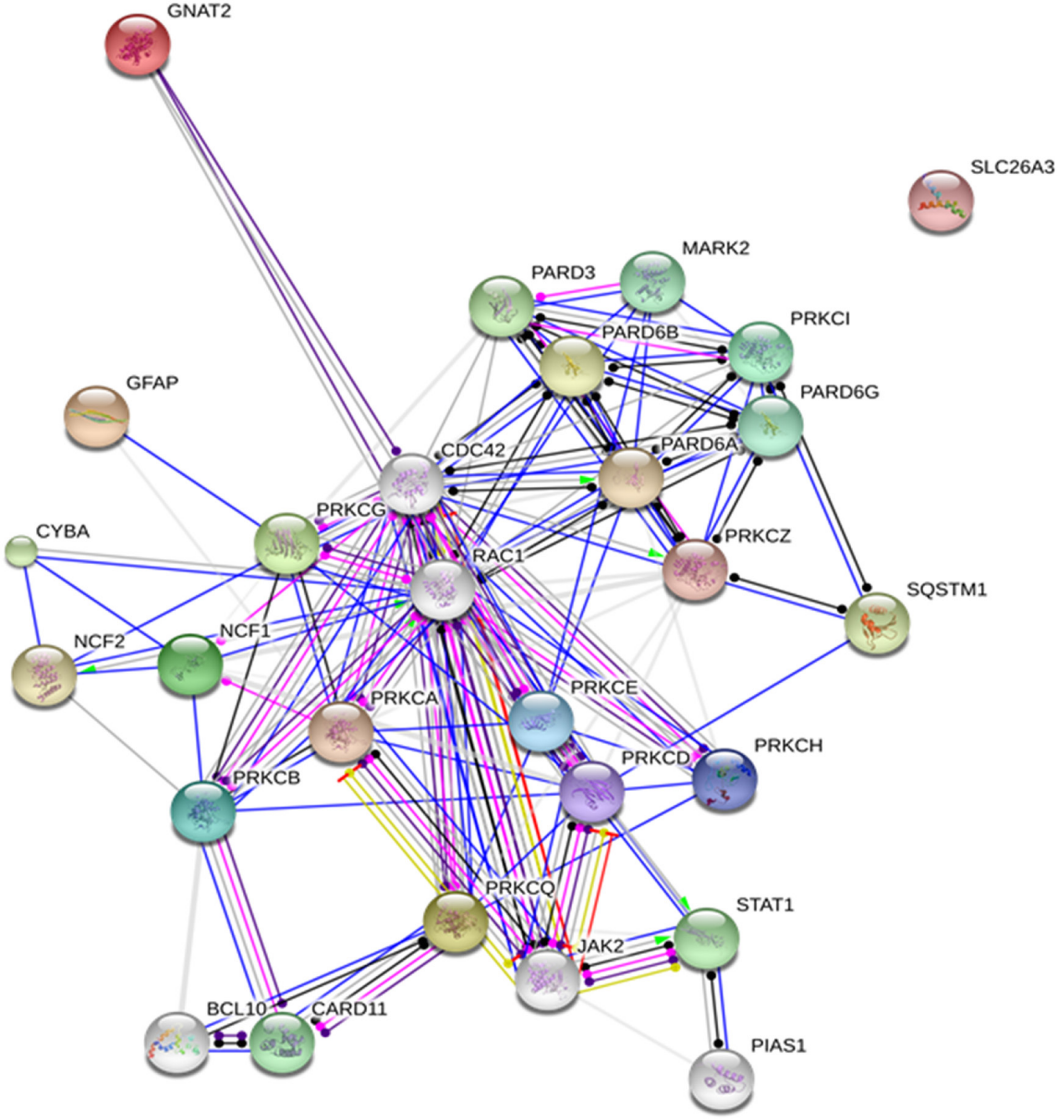
Protein network depicting interactions among protein kinase C variants and their potential substrates For generating the network the existing information of protein-protein interaction in the body of contemporary biomedical literature was exploited. Blue lines indicate direct binding between partnering molecules, green lines indicate activation and red lines indicate inhibition, whereas purple lines indicate catalysis, pink lines indicate posttranslational modification, yellow lines indicate transcriptional regulation and black lines indicate a generic reaction. Arrows indicate a positive action, bars indicate a negative action and ball-ends indicate a directional interaction of unknown nature. Proteins are represented by gene names. The network was generated by STRING 10.0 [19].

Subsequently we decided to further mine our data for relevant signaling events, especially in view of the relative unsuitability of PKC as a drug target. This can be done by challenging the data in the bioinformatical protein network generated by STRING 10.0 [19] using PKC variants as core proteins. Surprisingly a relevant involvement with JAK-STAT signaling in chemoresistant cells (Figure 5) emerged, prompting investigation of the role of this signaling in breast cancer chemoresistance.

### STATs are significantly up-phosphorylated in chemoresistant breast cancer cells

**Figure 6:**
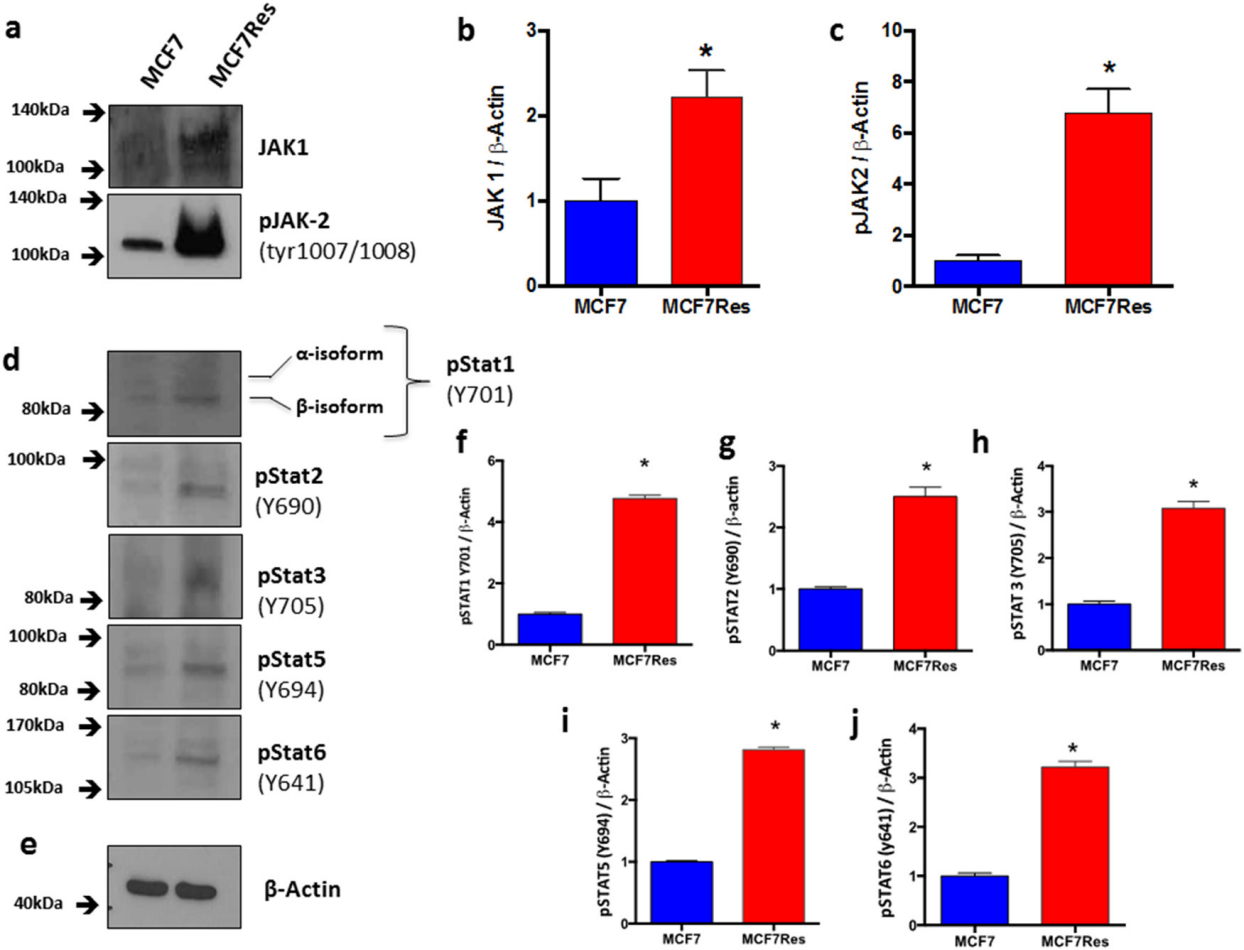
Differential JAK-STAT signaling involvement Cells were cultured under routine conditions. In the semi-confluence, the cells were lysed using standard lysing buffer (described in M&M), the whole protein was resolved on SDS-PAGE gel and after PVDF membrane transfer, these were identified by using specific primary antibodies. **(a)** Representative blottings for Jak1 and pJak2. Graphs **(b)** and **(c)** represents arbitrary values obtained by densitometric analysis of Jak1 and pJak2 bands normalized by the average values of the ß- Actin bands. Statistical analysis showed that effects with respect to Jak1 andJak2 were significant resistance. ß-Actin was used as loading control (approximately 75 μg of protein per lane). **(d)** Representative blotting for pStat1, pStat2, pStat3, pStat5 and pStat6. **(e)** Representative blotting for ß- Actin. Arbitrary values obtained by densitometric analysis and normalized for ß- Actin are shown as well **(f-j)**. Differences were considered significant when ^*^p<0.05.

In order to address the issue of JAK/STAT signaling in maintaining chemoresistance, we interrogated the phosphorylation status of various STATs including STAT1 (Y701) (Figure 6d, 6f), STAT2 (Y690) (Figure 6d, 6g), STAT3 (Y705) (Figure 6d, 6h), STAT5 (Y694) (Figure 6d, 6i) and STAT6 (Y641) (Figure 6d, 6j), as well as JAK1 and JAK2. All of the Stats investigated displayed significantly increased–phosphorylation in chemoresistant cancer cells. Likewise expression of both JAK1 and JAK2 were significantly upregulated in the chemoresistant phenotype (Figure 6a-6c), and especially phosphorylation of JAK2 was very prominent in this cell type, suggesting that the latter kinase is involved in maintaining the chemoresistant phenotype.

**Figure 7:**
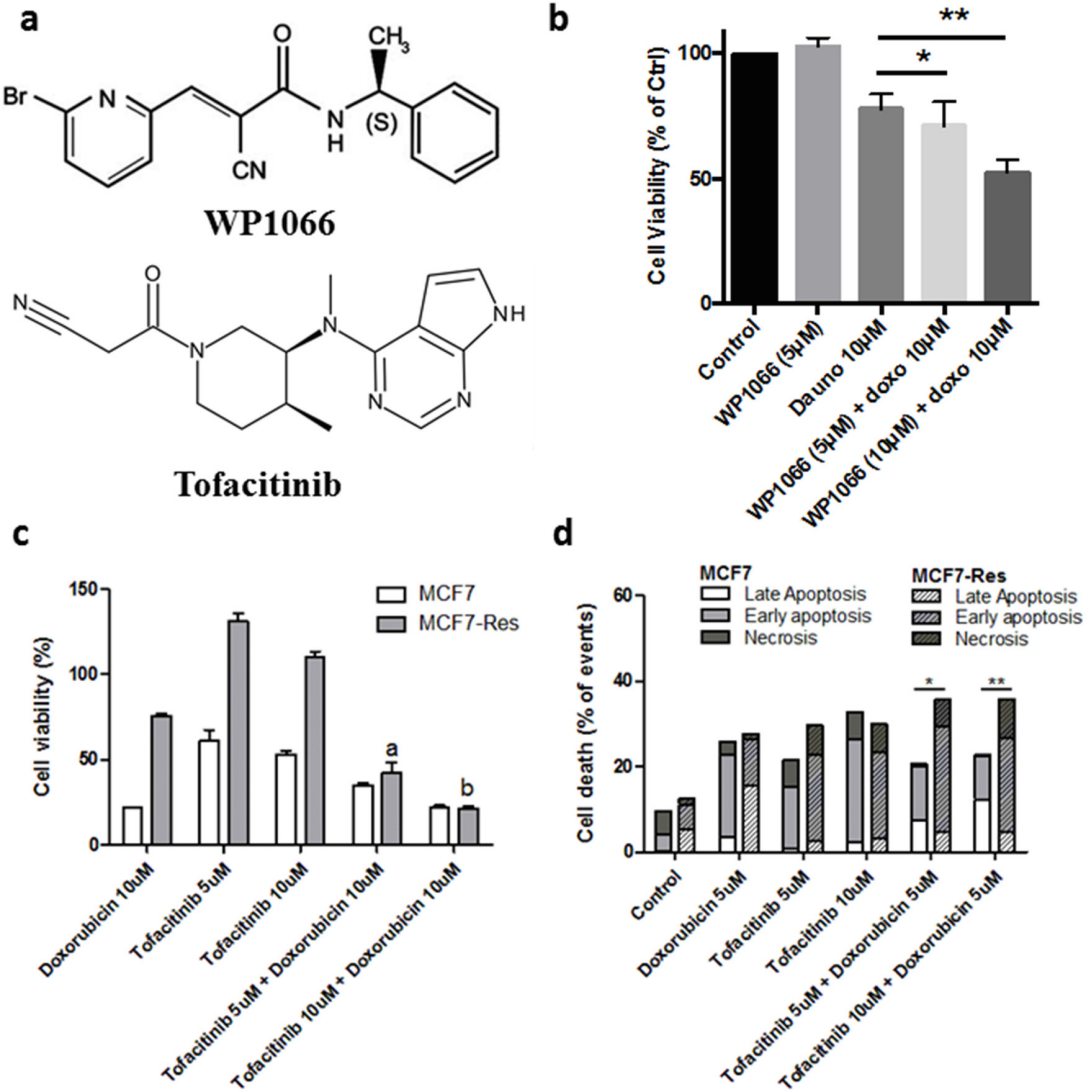
JAK inhibitor (Tofacitinib) sensitizes MCF7-Res cells to doxorubicin **(a)** Chemical structures of the WP1006 and Tofacitinib. **(b)** a Jak2 inhibitor sensitizes to chemotherapy in breast cancer cells. Cells were plated in 96 well plates and at the semi-confluence were treated with the concentrations of 5μM and 10μM inhibitor of Jak2 [(E)-3-(6-bromopyridin-2-yl)-2-cyano-N-((S)-1-phenylethyl) acrylamide, WP1066] for 2 hours. After this period, all the groups were treated with 10μM of doxorubicin for 24 hours, while the viable cells were monitored by MTT reduction capacity. Both concentrations significantly sensitized [5μM (^*^ p <0.05) and 10μM (^**^p <0.001)] cells initially resistant to doxorubicin (doxo). **(c)** Viable cells quantified by trypan blue. The MCF7-Res cells viability decrease in Tofacitinib and Doxorubicin treatment, indicated by 5μM and 10μM of Tofacitinib. Data are represented as mean ± S.E.M. **(d)** Flow cytometry to apoptosis assay. MCF7 and MCF7-Res cells were exposed to 5μM and 10μM of Tofacitinib for 2 hours; after treated with Doxorubicin 5μM for 24 hours. After treatment, the apoptosis was analyzed by a flow cytometer using annexin-V/7-AAD double-staining assay. Early apoptosis (annexin-V positive, 7-AAD negative), late apoptosis (double-positive cells), and non-apoptotic cell death (annexin-V negative, 7-AAD positive) are shown, as percentage. The difference of sensitivity to Doxorubicin monotherapy and co-therapy with Tofacitinib between MCF7 and MCF7-Res is indicated by (^*^) (5μM) and (^**^) (10μM) in favor for Tofacitinib co-therapy.

This notion was substantiated by experiments in which we tested the effect of two JAK2 inhibitors, [(E)-3-(6-bromopyridin-2-yl)-2-cyano-N-((S)-1-phenylethyl) acrylamide, WP1066] and FDA-approved Tofacitinib in the presence and absence of doxorubicin (Figure 7a) on the resistant breast cancer cells. WP1066 itself exerted no cytotoxicity at 5μM or 10μM. However, when cells were pretreated for two hours with 5μM or 10μM of the WP1066 and cells were subsequently challenged with doxorubicin for 24 hours, chemosensitivity of MCF7Res cell line was clearly evident [5μM (p<0.05) and 10μM (p<0.001)] (Figure 7b). Additionally, the same cellular response profile was observed in the case of combined treatment with Tofacitinib and doxorubicin (Figure 7c). Importantly, the drugs combination was more efficient in triggering late apoptosis (Figure 7d).

**Figure 8:**
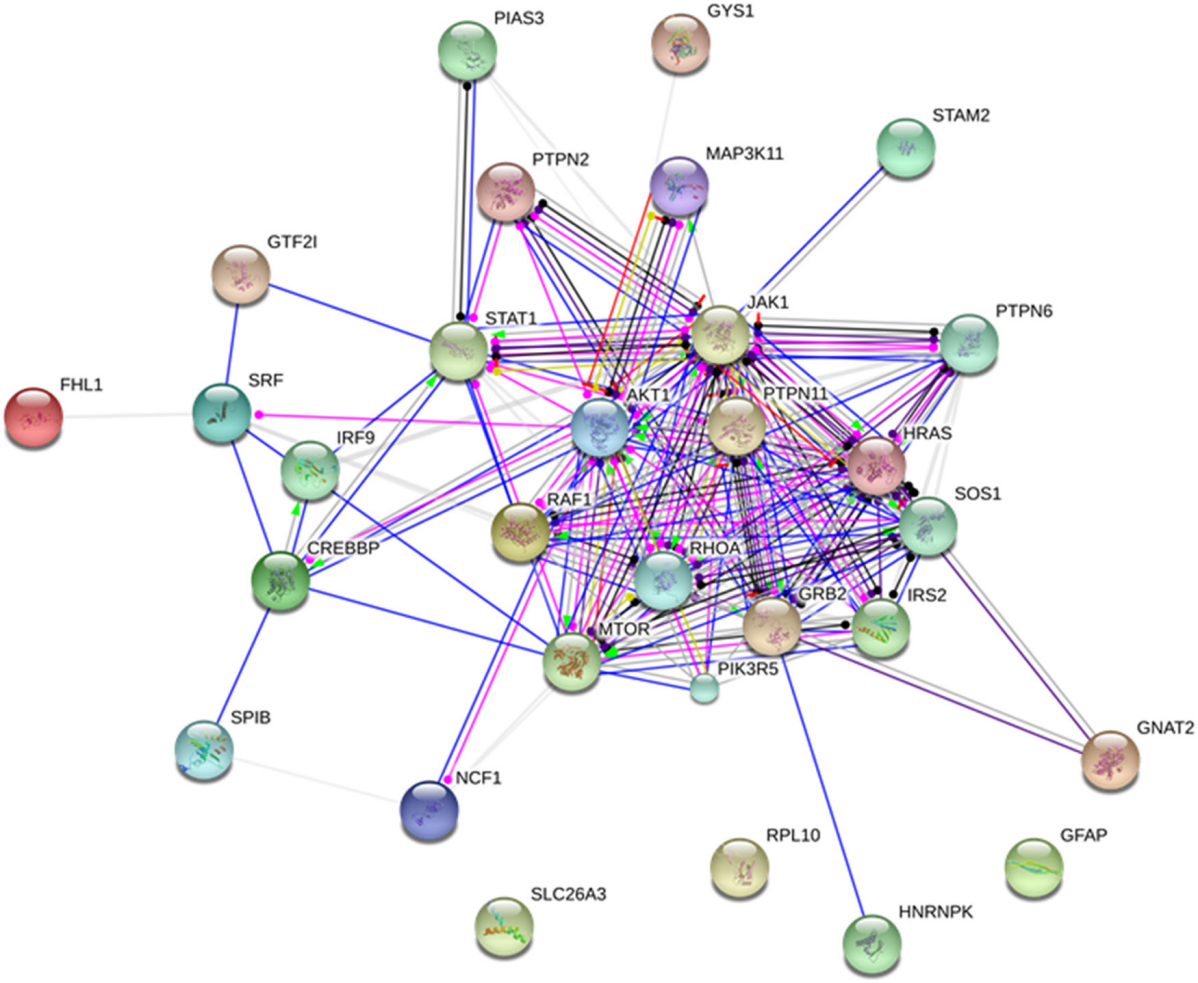
Protein network depicting interactions among Jak-Stat signaling pathway proteins (extracted from KEGG) and proteins found with altered phosphorylation profiles Blue lines indicate binding, green lines indicate activation and red lines indicate inhibition, whereas purple lines indicate catalysis, pink lines indicate posttranslational modification, yellow lines indicate transcriptional regulation and black lines indicate a generic reaction. Arrows indicate a positive action, bars indicate a negative action and ball-ends indicate a directional interaction of unknown nature. Proteins are represented by gene names. The network was generated by STRING 10.0 [19].

**Figure 9:**
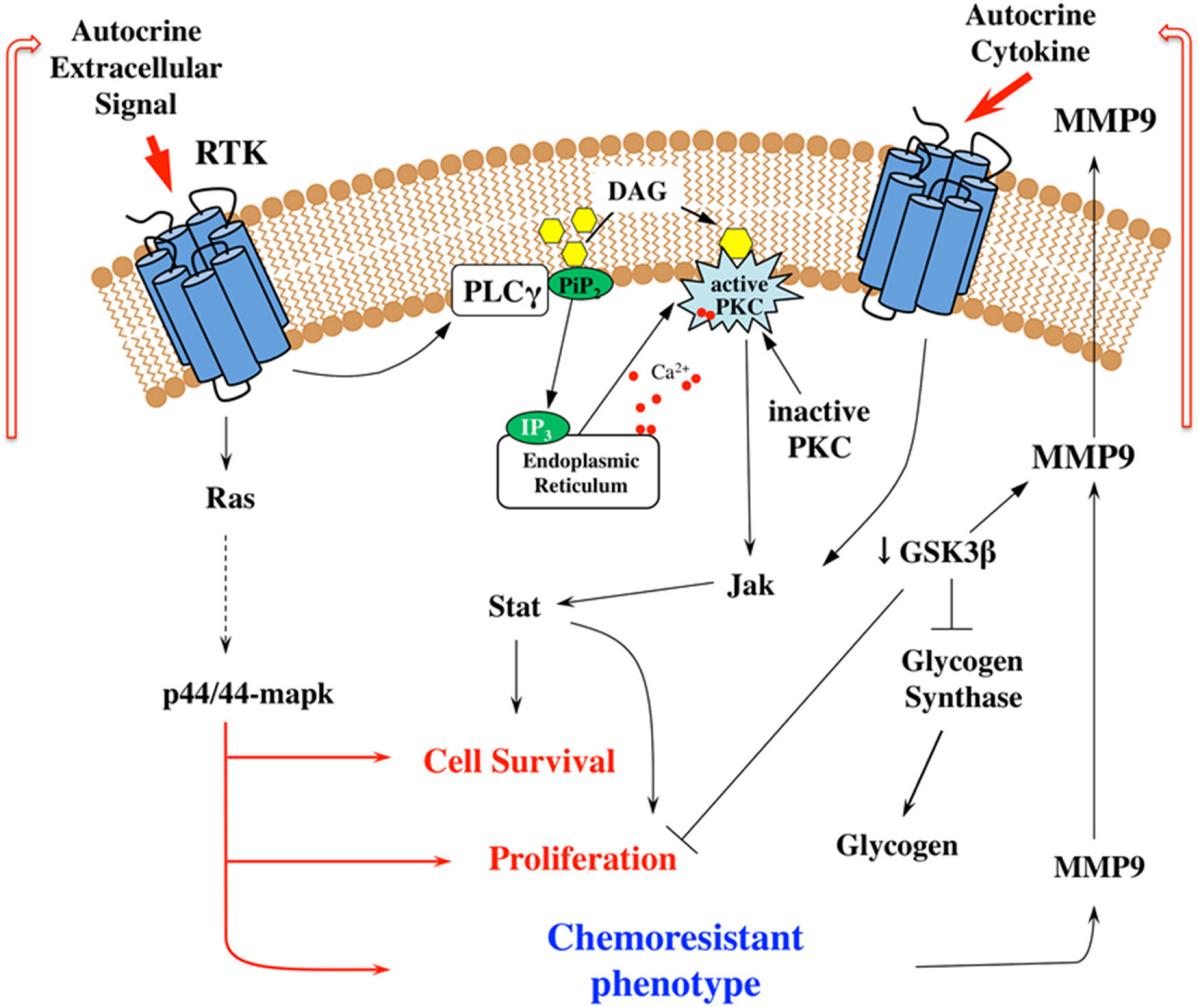
Signal transduction mechanisms involved in the resistance of breast tumor cells Schematic proposed mechanism of JAK-STAT signaling pathway and PKC activation and the relation to the acquisition of resistance to chemotherapeutic agents. The scheme shows elements actively investigated in this study and illustrates the potential to construct metabolic maps from kinome data.

In addition, the Figure 8 shows the protein network depicting interactions among JAK-STAT signaling pathway proteins (extracted from KEGG [20]) and proteins found with altered phosphorylation profiles in the phosphoproteomes obtained from breast cancer chemoresistant cells. Note the involvement of the JAK-STAT signaling with proteins related with cell proliferation (MAPKs, Raf-1), cytoskeleton rearrangement (RhoA), cell survival (PI3K, IRS, AKT1) and control of protein synthesis (mTOR). In conjunction, these data reveal a clear role for JAK-STAT signaling in maintaining the chemoresistant phenotype in breast cancer. The Figure 9 brings an overview of the relevant findings encountered in this study.

## DISCUSSION

One of the major obstacles in the treatment of cancer patients is to overcome resistance to chemotherapeutic agents. Therefore, the elucidation of mechanisms responsible to resistance to drugs with different chemical structures and targets in order to develop of new protocols for treating cancer-based disorders is very urgent called for. Here we tried to address this issue by contrasting kinome patterns in chemoresistant and chemosensitive MCF7 cultures. The results reveal that chemoresistance is associated with activation of classical PKCs but also that the chemoresistant phenotype is dependent on JAK2 signaling. As JAK2 is pharmaceutically relatively easy to target (*e.g*. the pan-JAK inhibitor tofacitinib is already routinely used for the treatment of inflammatory and neoplastic diseases [21]), these results strongly argue for clinical studies into the potential of JAK inhibitors to combat clinical chemoresistance in breast cancer. In addition, the multitude of kinases identified associated with the chemoresistant phenotype may also serve as a biomarker fingerprint for resistance development in the treatment of breast cancer.

Regarding the involvement of PKCs, an apparent paradox is present in our results: PKCαβII and PKD/PKCμ are differentially regulated as compared to PKCα and PKCδ in relation to the chemoresistant phenotype. The results correspond, however, with those of Koivunen et al. (2006) [22] who showed that PKCαβII phosphorylation positively correlates to a malignant phenotype in breast cancer. Likewise, overexpression of PKD/PKCμ correlates to chemoresistance in various cell types and is associated to increased proliferation and metastasis, possibly through activation of the PI3K-Akt and the Raf1-Mek-erk (p42/p44-MAPK) pathway [23]. We observed that activation of p42/p44-MAPK, overexpression of MMP-9 and downregulation of GSK3β in MCF7Res cells, which mirrors earlier observations of Kim et al. (2007) [24]. These authors showed that inhibition of GSK3β is necessary to maintain high MMP-9 levels through the activation of p42/p44-MAPK and a similar mechanism appears operative in MCFRes cultures. It is thus tempting to speculate that PKD/PKCμ contributes, together down-regulation of GSK3β and up-regulation of p42/p44-MAPK to the MDR-phenotype in breast cancer, concomitantly driving MMP-9 overexpression and thus metastasis. Such a scheme would fit well with earlier observations of higher PKD/PKCμ patterns in the MDR phenotype in breast cancer cells [25]. However, further studies remain necessary to prove this point.

Our observation of the strong activation of classical PKCs in chemoresistant breast cancer may help to bring clarity in a very controversial body of literature. At present current literature contains very contradictory results on the role of PKCα in chemoresistance, different groups reporting apparently contradictorily results. Importantly, though PKCα phosphorylation is usually increased in TAMR (tamoxifen-resistant) breast cancer cells when compared to TAMS (tamoxifen-sensitive) breast cancer cells [26]. In addition, it has been reported that PKCδ is a tumor suppressor protein [27] and also involved with metastatic progression [28]. We show PKC isoforms are highly differentially regulated with respect to chemoresistance in breast cancer cells, which may explain disparity in earlier results. In this context our results with regard to PLCγ expression may be relevant. PLCγ hydrolyzes phosphatidylinositol 4,5-bisphosphate to generate the second messengers, inositol 1,4,5-trisphosphate (IP3) and diacylglycerol (DAG). In turn, IP3 induces a transient release of intracellular free Ca^2+^ mainly from smooth endoplasmic reticulum, while DAG directly activates protein kinase C. Thus, traditional or conventional PKCs (cPKC) composed of PKCα, PKCβI, PKCβII and PKCγ are calcium-dependent and activated by phosphatidylserine (PS) and DAG. Our results show that PLCγ is overexpressed in MCF7Res cells and correlates to PKCβII phosphorylation. In view of the potential role of PLCγ in PKC activation, it has been suggested that its inhibition is an appropriate target to limit the metastatic potential of malignant cells [29–31]. Our results corroborate this notion. Generally speaking, however, PLC inhibitors with an acceptable toxicity profile are not available and thus clinical validation of this hypothesis cannot be done right now. In addition, if considering the critical roles of PKC signaling in both normal physiology and disease, even selectively targeting specific PKC isoforms may be associated with unacceptable toxicity and side effects.

In conclusion, we feel that our study provides a wealth of information on the biochemical events linked to development of the chemoresistant phenotype in breast cancer cells and especially implicates PKC and JAK/STAT signaling in this phenomenon. In view of the availability of approved JAK inhibitors, our results argue for clinical trials testing the potential of such inhibitors for combating breast cancer chemoresistance.

## MATERIALS AND METHODS

### Antibodies, chips and JAK inhibitors

The following antibodies were purchased from Cell Signaling (Danvers, MA, USA): JAK1 Antibody (#3332, 130kDa), Phospho-JAK2 (Tyr1007/1008) Antibody (#3771, 125kDa), β-Actin Antibody (#4967, 45kDa), Phospho-STAT1 (Tyr701) (58D6) Rabbit mAb (#9167, 84,91kDa), Phospho-STAT2 (Tyr690) Antibody (#4441, 113kDa), Phospho-STAT3 (Tyr705) Antibody (#9131, 79,86kDa), Phospho-STAT5 (Tyr694) Antibody (#9351, 90kDa), Phospho-STAT6 (Tyr641) Antibody (#9361, 110kDa), Ras Antibody (#3965, 21kDa), p44/42-MAPK (Erk1/2) Antibody (#9102, 42,44kDa), GSK-3β (27C10) Rabbit mAb (#9315, 46kDa), MMP-9 Antibody (#3852, 84,92kDa), Phospho-PKCα/β II (Thr638/641) Antibody (#9375, 80,82kDa); PKCα Antibody (#2056, 80kDa); Phospho-PKC (pan) (βII Ser660) Antibody (#9371, 78 a 85kDa); Phospho-PKD/PKCμ (Ser744/748) Antibody (#2054, 115kDa); PKD/PKCμ Antibody (#2052, 115kDa); PKCδ Antibody (#2058, 78kDa); Phospho-PKCδ/θ (Ser643/676) Antibody (#9376, 78kDa), PKCζ Antibody (#9372, 78kDa); PLCγ1 Antibody (#2822, 155kDa); anti-mouse, anti-rabbit and anti-goat IgGs antibodies. From Abcam (Cambridge, MA, USA): anti-PKC antibody (ab59363) was purchased. PepChip1-Kinomics^™^ slides were obtained from Pepscan Presto BV (Lelystad, the Netherlands).

WP1066 (#573097; C_17_H_14_BrN_3_O) and Tofacitinib (#PZ0017; C_16_H_20_N_6_O · C_6_H_8_O_7_) were purchased from Sigma-Aldrich (St. Louis, MO, USA).

### Cell line and culture conditions

The MCF7 cell line was purchased from the ATCC (ATCC, Manassas, VA, USA), and routinely cultured in DMEM medium supplemented with 10% FBS, 4mM glutamine, 1mM sodium pyruvate, 100 IU/mL penicillin and 100μg/mL streptomycin. Doxorubicin resistant variant cells (MCF7Res) derived from MCF7 cells were continuously cultured in the medium as described above containing additional 500nM doxorubicin (Sigma-Aldrich, St Louis, MO, USA), along with the parental MCF7-cells under identical culture conditions except that the control cells were treated with 0.1% ethanol. The two cell lines were grown side by side at all times. Cultures were maintained in 5% carbon dioxide at a temperature of 37°C.

### Cell viability was determined by MTT reduction

Both MCF7 and MCF7Res were plated into 96-well plates, 10,000 cell/well. Forty-eight hours later, the culture medium was replaced by fresh medium containing various concentrations of doxorubicin for 24h. The percentages of viable cells were then determined by the conversion of the water soluble MTT (3-(4,5-dimethylthiazol-2yl)-2,5-diphenyltetrazolium bromide) to an insoluble formazan, relative to drug free controls. All cytotoxicity data shown are the means of at least three independent experiments. Results of the MTT cell proliferation assay were analyzed using the GraphPad PRISM 6.0 software (GraphPad Software, Inc.). The inhibitory concentration (IC50) values, which are the drug concentration at which 50% of cells are viable, were calculated from the logarithmic trend line of the cytotoxicity graphs.

### Peptide microarray-based phosphoproteome

Kinome array analysis was done as described by Diks et al. [14] and van Baal et al. [15]. Furthermore, the protocol of the kinome array is described in detail on the Web site (http://www.pepscan.nl/pdf/Manual%20PepChip%20Kinase%200203.pdf). In short, cells were washed in PBS and lysed in a non-denaturing complete lysis buffer. The peptide arrays (Pepscan Presto BV), containing up to 1,024 different kinase substrates (in triplicate), were incubated with cell lysates for 2h in a humidified stove at 37°C plus ^33^P-γ-ATP. Subsequently, the arrays were washed in 2 M NaCl, 1% Triton-X-100, PBS, 0.1% Tween, and H_2_O, where after arrays were exposed to a phospho-imaging screen for 72 h and scanned on a phospho-imager (Fuji Storm 860, Stanford, GE). The density of the spots was measured and analyzed with array software.

### Immunoblotings

MCF7Res and MCF7 were routinely cultured and protein extracts were obtained using lysis cocktail (50 mM Tris [tris(hydroxymethy- l)aminomethane]–HCl [pH 7.4], 1% Tween-20, 0.25% sodium deoxycholate, 150 mM NaCl, 1 mM ethylene glycol tetraacetic acid (EGTA), 1 mM *O*-Vanadate, 1 mM NaF, and protease inhibitors [1 mg/ml aprotinin, 10 mg/ml leupeptin, and 1 mM 4-(2-amino- ethyl)-benzolsulfonyl-fluoride-hydrochloride]) for 2 h on ice, as used previously by de Souza Queiroz et al. (2007) [16] and de Fátima et al. (2008) [17]. After clearing by centrifugation, protein concentration was determined using the Lowry method [18]. An equal volume of 2-sodium dodecyl sulfate (SDS) gel loading buffer (100 mM Tris–HCl [pH 6.8], 200 mM dithiothreitol [DTT], 4% SDS, 0.1% bromophenol blue, and 20% glycerol) was added to samples and boiled for 5 min. Protein extracts were resolved by SDS–PAGE (10% or 12%) and transferred to PVDF membranes (Millipore). Membranes were blocked with either 1% fat-free dried milk or bovine serum albumin (2.5%) in Tris-buffered saline (TBS)–Tween-20 (0.05%) and incubated overnight at 48C with appropriate primary antibody at 1:1.000 dilutions. After washing in TBS–Tween-20 (0.05%), membranes were incubated with horseradish peroxidase- conjugated anti-rabbit, anti-goat, or anti-mouse IgGs antibodies, at 1:2.000 dilutions (in all immunoblotting assays), in blocking buffer for 1h. When it was necessary to investigate more than one protein on the same blot, stripping of the membranes was performed as recommended by Abcam (Cambridge, MA, USA), while taking special care to avoid contamination with traces of ß-mercaptoethanol, as this may damage antibody conformation. Detection was performed using enhanced chemiluminescence (ECL). For all Western blots ß-actin was used as an (additional) internal control.

### Phosphoproteome data analysis

Datasets from chips were generated by EisenLab ScanAlyze, version 2.50, array software, and then submitted to a statistical spot reliability approach called PepMatrix [32]. Data on each chip are generated in triplicate (*i.e*. the result of three different peptide phosphorylation reactions, as each peptide is present in triplicate). Intra-array variation with respect to identical phosphorylation of identical peptide as assessed for consistency using two indexes: standard deviation/average ratio (SD/A) and the ratio between average and median (A/M). Selected threshold parameters for this analysis were SD/A < 20% and 80% < A/M < 120%. Every spot that did not pass both these thresholds in any of the chips involved in this experimentation was rejected in not subjected to further analysis in our pipeline. The remaining data was studied according to standard statistical analysis and included calculation of fold change and t-tests (two-tailed, heteroscedastic) for identifying significantly different peptide phosphorylation between the experimental conditions (p < 0.05). Corresponding proteins represented by the oligopeptides used for substate phosphorylation were then analyzed using protein interaction data from databases such as HPRD [33] and UniProt (The UniProt Consortium) [34] to identify kinases and signaling pathways potentially affected in the different experimental conditions. Afterwards, altered kinase activities were validated by more specific methods, such as western blotting, and positive matches were studied in further detail as described herein.

### Functional approaches to estimate JAK/STAT signaling involvement on MCF7 chemoresistance

#### Effect of WP1066 in MCF7 resistance

To determine potential cytotoxicity of 5μM and 10μM Jak2/Stat3 inhibitor semiconfluent MCF7Res cells were treated for 24 hours with the drug and cell viability was assessed by MTT reduction. For measuring chemosensisitivity MCF7Res were plated in 96 well-plates and at the semi-confluence and treated with 5μM and 10μM WP1066 for 2 hours or 10μM of doxorubicin, for 24 hours. Subsequently, cultures were incubated with MTT vital dye and after 3 hours the viable cells were measured by solubilizing precipitate formazan from viable cells in DMSO, according to routine procedures.

#### Effect of FDA-approved Tofacitinib in MCF7 resistance

Before treatment, MCF7 and MCF7-Res cells (4×10^4^ cells/cm^2^) were plated in 96-well plates and allowed to recover for 24 hours. Subsequently, at semi-confluence, cells were treated with Tofacitinib 5μM and 10μM for 2 hours followed by a Doxorubicin 10μM challenge for 24 hours. Cell viability was assessed by trypan blue dye exclusion. Apoptosis was analyzed by annexin-V-APC binding and 7-AAD (BD Biosciences) exclusion in accordance with manufacturer’s instructions. Before treatment, MCF7 and MCF7-Res cells (4×10^4^ cells/cm^2^) were plated at 96-well plate and allowed to recover for 24 hours. At semi-confluence, cells were treated with 5μM and 10μM of Tofacitinib for 2 hours; and subsequently treated with Doxorubicin 5μM for 24 hours. After treatment, cells were harvested (Trypsin-EDTA 0.25 %) and resuspended at a density of 1×10^5^ cells in 100 μL of binding buffer (1X). Annexin-V-APC and 7-AAD were added to the cells and the suspension was incubated in the dark for 15 minutes at room temperature. After that, 200 μL of binding buffer was added to each tube. Cells were analyzed by a flow cytometer (FACS Canto II, BD Biosciences), 10,000 events. The results were analyzed as follows: live cells (double-negative cells), early apoptosis (annexin-V positive, 7-AAD negative), late apoptosis (double-positive cells), and non-apoptotic cell death (annexin-V negative, 7-AAD positive) fractions were established.

### Statistical analysis

Results are expressed as mean ± standard error of the mean (SEM). The statistical analyses were performed using analysis of variance (ANOVA) combined with appropriate Bonferroni correction. A p value <0.05 was considered to be statistically significant. The software used was GraphPad Prism 6.

## SUPPLEMENTARY MATERIALS FIGURES AND TABLES




