# Supplementary material for: Phosphoproteome profiling reveals critical role of JAK-STAT signaling in maintaining chemoresistance in breast cancer
Source: Oncotarget. 2017 Oct 10;8(70):114756–68. doi: 10.18632/oncotarget.21801 (PMC5777730; doi:10.18632/oncotarget.21801)
Supplement: Supplementary file 1 [file oncotarget-08-114756-s001.pdf]

Phosphoproteome profiling reveals critical role of JAK-STAT signaling in maintaining chemoresistance in breast cancer

SUPPLEMENTARY MATERIALS

Supplementary Table 1: Significantly differentially phosphorylated consensus peptides

See Supplementary File 1
